# À propos d’un hémangiome du genou

**DOI:** 10.11604/pamj.2017.27.225.12341

**Published:** 2017-07-27

**Authors:** Hicham Bousbaa, Larbi Amhajji

**Affiliations:** 1 Department of Orthopaedics and Traumatology, Military Hospital Moulay Ismail, BP 50000 Meknes, Morocco

**Keywords:** Genou, hémangiome musculaire, IRM, Knee, muscle hemangioma, MRI

## Abstract

Angioma or hemangioma is a benign vascular tumor which rarely occurs in muscles. It mostly affects young adults. We report a case of intramuscular hemangioma of quadriceps with painless extension contracture of the knee and a mass in the thigh. MRI allowed preoperative diagnosis of intramuscular hemangiomas and, primarily, T2-weighted images allow specific topographic assessment and give an indication to the diagnosis.

## Image en médecine

L'hémangiome est une malformation vasculaire encore appelée angiome immature. Il s'agit d'une prolifération de capillaires immatures et de cellules épithéliales. Ce sont des tumeurs vasculaires bénignes mais potentiellement graves selon le siège, dont la localisation musculaire est rare. Une jeune femme de 46 ans était adressée à la consultation, pour l'apparition d'une masse douloureuse, localisée à la partie inféro-interne de la cuisse droite. L'évolution a été marquée par l'augmentation progressive de la taille depuis 1 an et l'aggravation de la douleur devenant gênante de plus en plus lors des activités sportives. L'examen clinique retrouve une tuméfaction mobile, légèrement indurée à la palpation le reste de l'examen sans particularités. La radiographie standard était normale. L'imagerie par résonnance magnétique du genou droit montrant un processus lésionnel du muscle vaste interne au niveau du tiers inférieur en hyposignal T1 (A) et en hypersignal T2 (B) présentant une extension intra articulaire au niveau de la bourse sous quadripitale rehausse de façon importante et hétérogène après injection intraveineuse du produit de contraste (C) évoquant un angiome musculaire. Après exérèse totale l'anatomo-pathologiste a confirmé la nature de la tumeur.

**Figure 1:**
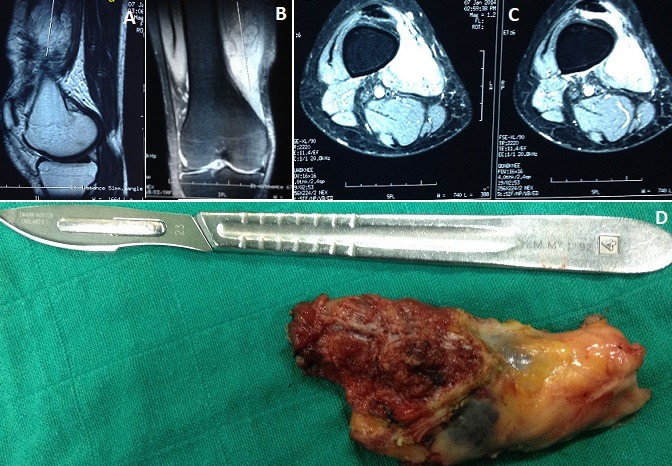
l'imagerie par résonnance magnétique du genou droit montrant un processus lésionnel du muscle vaste interne au niveau du tiers inférieur en hyposignal T1: (A) et en hypersignal T2; (B) présentant une extension intra articulaire au niveau de la bourse sous quadripitale rehausse de façon importante et hétérogène après injection intraveineuse du produit de contraste; (C) évoquant un angiome musculaire; (D) la pièce opératoire

